# Thermal Delamination Modelling and Evaluation of Aluminium–Glass Fibre-Reinforced Polymer Hybrid

**DOI:** 10.3390/polym13040492

**Published:** 2021-02-04

**Authors:** Zhen Pei Chow, Zaini Ahmad, King Jye Wong, Seyed Saeid Rahimian Koloor, Michal Petrů

**Affiliations:** 1 School of Mechanical Engineering, Faculty of Engineering, Universiti Teknologi Malaysia, Johor Bahru 81310, Malaysia; zpchow2@graduate.utm.my (Z.P.C.); kjwong@mail.fkm.utm.my (K.J.W.); 2 Institute for Nanomaterials, Advanced Technologies and Innovation (CXI), Technical University of Liberec (TUL), Studentska 2, 461-17 Liberec, Czech Republic; seyed.rahimian@tul.cz (S.S.R.K.); michal.petru@tul.cz (M.P.); 3 Department of Aerospace Engineering, Faculty of Engineering, Universiti Putra Malaysia, Serdang 43400, Malaysia

**Keywords:** metal–composite hybrid laminate, finite element analysis, temperature effect, cohesive zone modelling, Mode-I delamination, Mode-II delamination

## Abstract

This paper aims to propose a temperature-dependent cohesive model to predict the delamination of dissimilar metal–composite material hybrid under Mode-I and Mode-II delamination. Commercial nonlinear finite element (FE) code LS-DYNA was used to simulate the material and cohesive model of hybrid aluminium–glass fibre-reinforced polymer (GFRP) laminate. For an accurate representation of the Mode-I and Mode-II delamination between aluminium and GFRP laminates, cohesive zone modelling with bilinear traction separation law was implemented. Cohesive zone properties at different temperatures were obtained by applying trends of experimental results from double cantilever beam and end notched flexural tests. Results from experimental tests were compared with simulation results at 30, 70 and 110 °C to verify the validity of the model. Mode-I and Mode-II FE models compared to experimental tests show a good correlation of 5.73% and 7.26% discrepancy, respectively. Crack front stress distribution at 30 °C is characterised by a smooth gradual decrease in Mode-I stress from the centre to the edge of the specimen. At 70 °C, the entire crack front reaches the maximum Mode-I stress with the exception of much lower stress build-up at the specimen’s edge. On the other hand, the Mode-II stress increases progressively from the centre to the edge at 30 °C. At 70 °C, uniform low stress is built up along the crack front with the exception of significantly higher stress concentrated only at the free edge. At 110 °C, the stress distribution for both modes transforms back to the similar profile, as observed in the 30 °C case.

## 1. Introduction

Fibre metal laminates (FMLs) are increasingly used in aeronautical and marine applications for their combination of advantages from two different materials. FMLs involve adhesively bonding alternating metal and composite layers to form a laminated sandwich structure. While pure composite materials are prone to brittleness, temperature effects and barely visible internal damage, pure metals are susceptible to fatigue loading and lower yield strength. However, the hybrid nature of the FML allows beneficial properties from both metal and composite counterparts, including lighter weight, ductility, fatigue resistance and damage tolerance [1,2,3]. The multiple layers of the FML structure prevent crack propagation from damaged layers to subsequent layers via crack bridging and prevent catastrophic failure that would otherwise occur unimpeded in monolithic materials. Similarly, multiple alternating laminates are capable of suppressing the spread of thermal and moisture absorption across the layers that allow potential applications of FMLs in adverse conditions [3,4]. However, laminating sheets of dissimilar materials into a single component requires an adhesive agent. As such, the adhesive part between hybrid metal–composite materials, in turn, becomes the weaker part of the laminate, prone to separation [5,6]. Failures in laminate adhesives primarily include Mode-I tensile peeling and Mode-II shearing delamination.

Finite element (FE) numerical simulation can be a very useful tool to predict and recreate the responses of delamination, and at the same time provide greater understanding and visualisation of delamination mechanisms. Cohesive zone models have been used with the bilinear traction separation law and delamination criteria to simulate different modes of delamination with good accuracy [7,8,9,10,11]. Turon et al. [7] developed an effective method of simulating composite delamination with larger elements to allow reduced computational time by establishing analytical equations for choosing penalty stiffness and a standard for reducing peak traction stresses while increasing cohesive length. Moreover, the approach was expanded upon for mixed-mode loading in their subsequent research where it is discovered that relations between interlaminar strength and stiffness based on their method are capable of obtaining results in any mode ratio of loading [8]. A cohesive zone model capable of simulating mixed-mode quasi-static and fatigue growth delamination after exposure to moisture content was contributed by LeBlanc and LaPlante [9]. Johar et al. [10] developed a methodology to estimate Mode-II delamination properties of composite laminates by adopting data reduction and classical laminate theory with finite element simulation. Using a combined experimental and numerical method, Koloor and Tamin [11] scrutinised the delamination crack growth process of carbon fibre-reinforced polymer (CFRP).

Simulation of FML delamination can prove to be a challenging task as they have separate material properties. The bending and flexural performance of glass laminate aluminium-reinforced epoxy (GLARE) with different varieties of layups were investigated by Li et al. [12] with combined experimental and numerical finite element analyses. Due to the ductile nature of metals, the fracture energy of the metal–composite interface is higher than the actual interface energy due to metal plasticity from bending in Mode-I [13]. An in-depth finite element analysis on Mode-I and mixed-mode I/II by Zhao et al. [14] found that the interface strength of composite laminates within a certain boundary has minuscule effects on the simulation results. This insignificant effect of interface strength is also stated by Delbariani et al. [15] in their study of metal–composite joints. Tsokanas and Loutas [16] expressed the complications in analytical modelling of dissimilar materials due to disparate ductility and thermal behaviour which have substantial effects on delamination properties. Hence, the thermomechanical properties of each material component in a hybrid material should be represented and modelled separately to yield accurate analytical results.

There is an abundance of literature on the hygrothermal effects on delamination, which determines the delamination properties of pure composite and dissimilar materials after ensuing thermal and moisture effects [16,17,18]. On the other hand, there are also plenty of authors who investigated temperature effects on pure composites. Cadieu et al. [19] studied the effects of loading rate and temperature on composite thermoplastic laminates under Mode-I delamination, where they discovered that temperature below room temperature has almost no effect on fracture toughness. The effects of temperature ranging from –43 °C to 125 °C and moisture on delamination of carbon/epoxy composites were investigated by Davidson et al. [20]. They discovered improvement of Mode-I but a deterioration of Mode-II toughness with increasing temperature.

A modified thermal inclusive cohesive zone model was applied and validated by Ibrahim and Albarbar [21] by comparing their model results with literature data on composite laminates ranging from the lowest temperatures of –100 °C to the highest of 150 °C. The thermal expansion coefficient and effect of temperature parameters based on the Helmholtz free energy equation were included in the damage evolution law of cohesive zone elements. Moreover, fracture energy varies with temperature based on the square of peak traction stresses and interface penalty stiffness were estimated from the literature. On the other hand, Gunther et al. [22] developed a temperature-dependent cohesive zone model for bi-metal laminates from 23 to 150 °C by performing tubular butt joint tests both in tension and torsion setups, a tapered double cantilever beam and tapered end notched flexure tests. Tubular butt joint tests were performed to extract adhesive peak loads with the means of calculating cohesive peak traction stresses, while concurrently obtaining penalty stiffness from 10% of the total strain.

There are only a few known studies on delamination with simultaneous temperature effects on dissimilar laminates. Qin et al. [23] experimentally and numerically researched the effects of temperatures at −40, 23 and 80 °C on a variety of adhesive joints between CFRP and aluminium. They applied fitting quadratic stress criteria to obtain cohesive failure stresses, estimated the fracture toughness values from the literature and assumed that the ratio of Young’s and shear moduli to be the same across different temperatures. Fibre metal laminates will continue to be prevalent in broad ranges of vehicles, including exposure to high temperatures. A methodology is required to procedurally obtain and effectively analyse the Mode-I and Mode-II delamination properties between dissimilar materials of FMLs at higher temperatures. The findings aid in ensuring proper suitability of materials and adhesives based on different applications.

In this study, the temperature effects on Mode-I and Mode-II delamination of hybrid glass fibre-reinforced polymer laminated with aluminium alloy are numerically investigated. Explicit nonlinear code LS-DYNA is employed to construct a finite element model of the experimental double cantilever beam (DCB) and end notched flexural (ENF) tests. The adhesive interface is modelled based on cohesive zone modelling defined with the bilinear traction separation law. The main cohesive parameter, fracture energy, is characterised by data reduction of experimental DCB and ENF load–displacement results by using the Irwin-Kies equation. The finite element models are then compared and validated with the experimental tests. Temperature effects on the stress distribution of the delamination crack front are investigated through validated FE models.

## 2. Material Modelling Constitutive Equations

Materials of the FML hybrid comprise metal and a composite part which, in the current study, are aluminium 2024-T3 and glass fibre-reinforced polymer (GFRP), respectively. Dissimilar mechanical properties require separate material modelling to accurately capture their behaviour and changes across different temperatures. A cohesive zone model is used to represent delamination between aluminium and GFRP.

### 2.1. Aluminium Material Modelling

The aluminium part is modelled with a Johnson–Cook material model that can sufficiently capture the effects of temperature. Flow stress, *σ_y_*, is expressed as [24]:

(1) $$\sigma_{y}=\left(A+B{\bar{\varepsilon }}_{p}{}^{n}\right)\left(1+C\ln\frac{\dot{\varepsilon }}{{\dot{\varepsilon }}_{0}}\right)\left[1-{\left(\frac{T-T_{room}}{T_{melt}-T_{room}}\right)}^{m}\right]$$

where *A*, *B* and *n* are the plastic strain material constants, ${\bar{\varepsilon }}_{p}$ is the effective plastic strain, *C* is the strain rate effect constant, $\dot{\varepsilon }$ is the effective total strain rate, ${\dot{\varepsilon }}_{0}$ is the quasi-static threshold rate, *m* is the temperature effect constant, *T* is the effective room temperature, *T_room_* is the room temperature and *T_melt_* is the melting point of the aluminium. The fracture strain with the effect of pressure, strain rate and temperature is:

(2) $$\varepsilon_{D}^{pl}=\left(d_{1}+d_{2}e^{-d_{3}\eta }\right)\left(1+d_{4}\ln\frac{\dot{\varepsilon }}{{\dot{\varepsilon }}_{0}}\right)\left[1+d_{5}\left(\frac{T-T_{room}}{T_{melt}-T_{room}}\right)\right]$$

where *d_1_*, *d_2_* and *d_3_* are the pressure effect constants, η is the ratio between pressure and effective stress, *d_4_* is the strain rate effect constant and *d_5_* is the temperature effect constant.

### 2.2. GFRP Material Modelling

On the other hand, GFRP is modelled using Chang–Chang failure criteria. Each failure mode is initiated accordingly depending on whether each of its corresponding equation shown below has been met [25]. However, since the Chang–Chang model does not include temperature effects, GFRP material properties at each temperature are included accordingly based on the respective temperature.

For the tensile fibre mode:

(3) $$\sigma_{1}>0\;\;\;then\;\;\;e_{f}{}^{2}={\left(\frac{\sigma_{1}}{X_{T}}\right)}^{2}+\beta {\left(\frac{\tau_{12}}{S_{C}}\right)}^{2}-1\{\begin{array}{c} \geq 0\;failed\\ <0\;elastic \end{array},\;E_{A}=E_{B}=G_{AB}=\nu_{BA}=\nu_{AB}=0$$

For the compressive fibre mode:

(4) $$\sigma_{1}<0\;\;\;then\;\;\;e_{c}{}^{2}={\left(\frac{\sigma_{1}}{X_{C}}\right)}^{2}-1\{\begin{array}{c} \geq 0\;failed\\ <0\;elastic \end{array},\;E_{A}=\nu_{BA}=\nu_{AB}=0$$

For the tensile matrix mode:

(5) $$\sigma_{2}>0\;\;\;then\;\;\;e_{m}{}^{2}={\left(\frac{\sigma_{2}}{Y_{T}}\right)}^{2}+{\left(\frac{\tau_{12}}{S_{C}}\right)}^{2}-1\{\begin{array}{c} \geq 0\;failed\\ <0\;elastic \end{array},\;E_{B}=\nu_{BA}=0\;\to G_{AB}=0$$

For the compressive matrix mode:

(6) $$\sigma_{2}<0\;\;\;then\;\;\;e_{d}{}^{2}={\left(\frac{\sigma_{2}}{2S_{C}}\right)}^{2}+\left[{\left(\frac{Y_{C}}{2S_{C}}\right)}^{2}-1\right]\frac{\sigma_{2}}{Y_{C}}+{\left(\frac{\tau_{12}}{S_{C}}\right)}^{2}-1\{\begin{array}{c} \geq 0\;failed\\ <0\;elastic \end{array},\;E_{B}=\nu_{BA}=\nu_{AB}=0\;\to G_{AB}=0$$

where *σ_1_* and *σ_2_* represent the effective stress tensor components in respective directions, *τ_12_* represents the effective shear tensor, *X_T_* and *X_C_* are the longitudinal tensile and compressive strengths, *Y_T_* and *Y_C_* are the transverse tensile and compressive strengths, *S_C_* is the shear strength, *E_A_* and *E_B_* are the longitudinal and transverse Young’s modulus, *G_AB_* is the shear modulus, *υ_BA_* and *υ_AB_* are the Poisson’s ratio in respective directions, and *β* is the weighting factor for shear term.

### 2.3. Cohesive Zone Modelling

**Figure 1:**
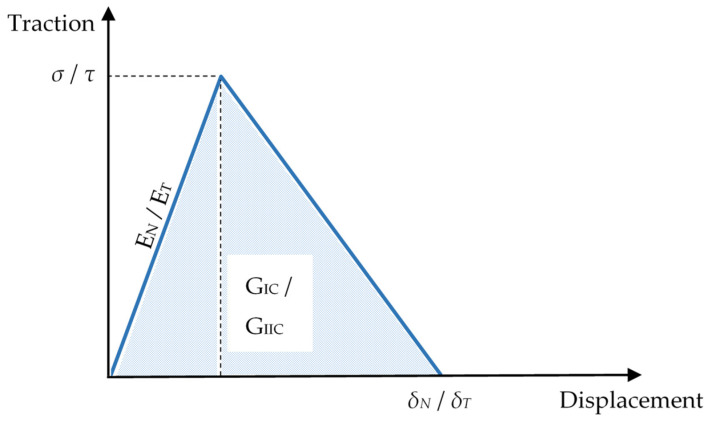
Bilinear traction separation law.

The interface is defined as 8-point cohesive elements with the same thickness of 13 µm as the Teflon material in the laminate pre-crack. By implementing the bilinear traction separation law in the cohesive elements, the process of delamination can be captured correctly. As shown in Figure 1, delamination behaviour, including increasing traction forces, followed by reaching the peak of interface strength, then the degradation of the interface and finally the full separation of the laminate can be captured by the traction separation law. Similar to Chang–Chang failure, the cohesive model implemented does not capture temperature effects; hence, the inputs of each temperature are defined separately. To ensure proper stress transfer between cohesive and neighbouring material, the cohesive elements are aligned to their adjacent material elements with the same mesh size to share the same node positions. Subsequently, the coinciding nodes between the cohesive and adjacent material nodes are merged into a single node.

The Mode-I energy release rate is defined as [26]:

(7) $$G_{IC}=\sigma \times \frac{\delta_{N}}{2}$$

where σ and *δ_N_* are the normal direction peak traction and ultimate displacement. *E_N_* is defined as the Mode-I penalty stiffness.

The Mode-II energy release rate is defined as [26]:

(8) $$G_{IIC}=\tau \times \frac{\delta_{T}}{2}$$

where *τ* and *δ_T_* are the tangential direction peak traction and ultimate displacement. *E_T_* is defined as the Mode-II penalty stiffness.

## 3. Finite Element Modelling 

Commercial LS-DYNA/Explicit software is used in this research to develop finite element models of Mode-I and Mode-II delamination with a combination of material models for aluminium and GFRP and a cohesive zone model. Experimental tests were conducted beforehand, where they are reported thoroughly in previous literature [27,28], with dimensions, materials and boundary conditions matching the finite element model. The results are then compared with numerical predictions to validate the models. 

Constant stress solid elements are used to define the aluminium and GFRP parts. Aluminium 2024-T3 mechanical properties based on the Johnson–Cook material model are shown in Table 1, while the material properties of GFRP previously extracted from experimental tests at each temperature are in Table 2. Base material properties of aluminium, including density, Young’s modulus, shear modulus and Poisson’s ratio values, are acquired from Wen et al. [29]. The Johnson–Cook yield surface parameters and failure parameters are adopted from Lesuer [30] and Li et al. [31]. Material properties of GFRP at each temperature are adopted from previous work [28].

**Figure 2:**
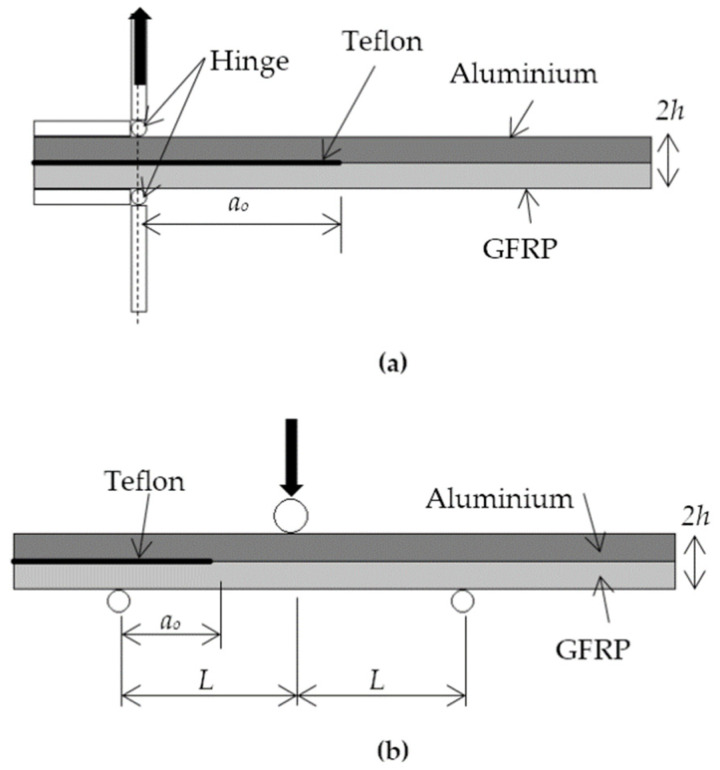
Setup of (**a**) Mode-I and (**b**) Mode-II delamination.

According to Figure 2, Mode-I delamination is modelled based on DCB while Mode-II is based on ENF experimental setups, as described in depth in previous publications [27,28]. Based on DCB and ENF experiments, the thickness of GFRP is modelled as 2.2 mm while aluminium is 2 mm. The primary fibre direction of GFRP is along the specimen length. The thickness of the main cohesive elements is modelled based on the thickness of nonadhesive Teflon in the experimental test, 13 µm. Hence, the total thickness of the hybrid model, *2 h*, is 4.213 mm. The total length and width for the Mode-I model are 125 mm × 20 mm, while for Mode-II, the dimensions are 175 mm × 20 mm. Both Mode-I and Mode-II specimens have fixed crack lengths *a_o_* of 40 and 25 mm, respectively. The span length for the ENF test, *L*, is 50 mm. The results from experiments are shown in Table 3, where the slope, *k*, and peak loads, *F_P_*, are used in data reduction to obtain fracture toughness. 

For the DCB model, shell elements are used to model the plates of hinges. Based on Figure 2a, horizontal plates are fixed to the surface of the laminate while being hinged with vertical plates. Therefore, the edges of two shell planes perpendicular to each other are joined and constrained, permitting only revolutions along the joined tip axis to simulate the rotating hinge. The bottom vertical plate is fixed to disallow any movement or rotation, while the top vertical plate is only allowed to move in the axial direction where it pulls the joined horizontal plate via the hinge. On the other hand, the 3-point bending loading is modelled using 3 rollers, defined as solid elements, as shown in Figure 2b. Two bottom rollers are fixed while the top roller can only move downwards along the vertical direction, as shown. 

The normal penalty stiffness, *E_N_* is calculated from [10]:

(9) $$E_{N}=\frac{E}{t}$$

where *E* is Young’s modulus of the adhesive with 6.03 GPa and *t* is the adhesive thickness at 0.013 mm.

The in-plane penalty stiffness, *E_T_*, is calculated from [10]:

(10) $$E_{T}=\frac{G}{t}$$

where *G* is the shear modulus of the adhesive at 0.90 GPa.

Based on the Irwin-Kies data reduction equations, slope, *k*, and peak load, *F_P_*, from Table 3 are converted into the respective fracture toughness required for cohesive parameters at each temperature. Peak traction stresses, *σ* and *τ*, are estimated empirically from compilations of previous literature on Mode-I and Mode-II interface strength done by Zhao et al. [14], where the values are similar to a literature review of aluminium and GFRP laminates. To acquire the cohesive properties at higher temperatures, a ratio approach between experimental data and cohesive properties was implemented in this study. 

The values of penalty stiffness, *E_N_* and *E_T_*, at higher temperatures are ratioed to the ratio of the slope, *k*, from a load–displacement curve based on Equations (11) and (12) [32].

(11) $$E_{N\left(x\right)}=\frac{k_{I\left(x\right)}}{k_{I\left(30\;{}^{\circ}C\right)}}\times E_{N\left(30\;{}^{\circ}C\right)}$$

(12) $$E_{T\left(x\right)}=\frac{k_{II\left(x\right)}}{k_{II\left(30\;{}^{\circ}C\right)}}\times E_{T\left(30\;{}^{\circ}C\right)}$$

where *k_I_* is the slope for Mode-I and *k_II_* is the slope for Mode-II; *x* is the respective temperature based on the required parameter.

On the other hand, the value of peak traction stresses is ratioed concerning the fracture toughness ratio between different temperatures based on Equations (13) and (14), where a similar method was used by Qin et al. [23] to acquire low and elevated temperature properties.

(13) $$\sigma_{\left(x\right)}=\frac{G_{IC\left(x\right)}}{G_{IC\left(30\;{}^{\circ}C\right)}}\times \sigma_{\left(30\;{}^{\circ}C\right)}$$

(14) $$\tau_{\left(x\right)}=\frac{G_{IIC\left(x\right)}}{G_{IIC\left(30\;{}^{\circ}C\right)}}\times \tau_{\left(30\;{}^{\circ}C\right)}$$

The resulting cohesive parameters at each temperature are shown in Table 4.

To simulate the quasi-static rate of loading with minimal kinetic effect and vibrations, the rate of loading must be set as low as possible. The top plate of the DCB simulation is set to move upwards at an increasing rate up to a maximum of 1 m/s. On the other hand, the ENF top roller moves downward at an increasing rate up to a maximum of 0.5 m/s. The loading rate at the time of crack initiation below 0.5 m/s ensures minimal effects on the cohesive properties [19]. To minimise computational time, the elements are reduced by using half models since both cases are symmetrical across the length. Boundary constraints are utilised with elements along the cut section of the half models. To control the hourglass effects of elements, Flanagan–Belytschko stiffness for hourglass control is defined for the aluminium.

A mesh convergence study is conducted by comparing the load–displacement curve of the FE model to experimental results at room temperature. It was found that an element size of 0.5 mm was able to produce good results within acceptable computational time. Mass scaling is applied for the models within an acceptable range with negligible dynamic effects to increase the simulation time step and reduce the total simulation run time.

## 4. Numerical Results and Analysis

The following section discusses the validation and analysis of Mode-I and Mode-II delamination models at temperatures of 30, 70 and 110 °C. A mesh convergence study is highlighted in Section 4.1 to demonstrate the optimum mesh size that is capable of accurately capturing the delamination mechanism. In Section 4.2, validation is carried out using load–displacement curves obtained from the results. Slopes and peak load between experimental and simulation results are compared to demonstrate the feasibility of models by percentage agreements. Section 4.3 elaborates on the localised stress distribution along the crack tip and how temperature affects this stress. The stress contour shows how stress builds up along the crack front and location of propagation while a detailed plot of mode stress versus distance from the centre of the laminate is used to describe it in detail.

### 4.1. Mesh Convergence

The fundamental process of the mesh convergence study is carried out during FE modelling to ensure the model can produce accurate results. The element size within a model affects the model accuracy where a large mesh size captures exceedingly large stresses. By refining into smaller mesh sizes, the effects of discretisation can be reduced, thus producing a solution closer to real-life specimen counterparts. However, a large mesh number increases the FE simulation time. With a sufficiently fine mesh, the FE model will be able to produce accurate results where further refinements have negligible improvements, therefore preventing the need for finer mesh than optimum where simulation time becomes unnecessarily long.

Mesh sizes of increasing refinement of 10, 5, 2, 1, 0.5 and 0.25 mm were modelled for Mode-I. A similar material model, element type and boundary conditions were defined for all of the mesh sizes. The load–displacement curve of each mesh size is then plotted and compared with the experimental results.

**Figure 3:**
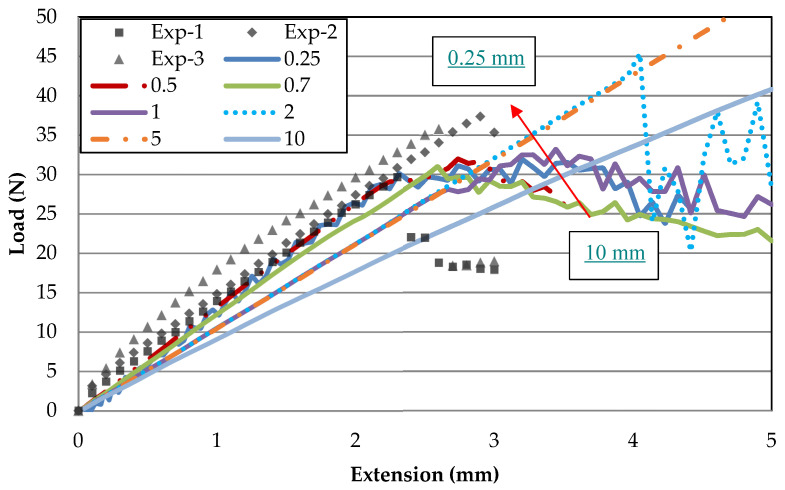
Mesh size effect on Mode-I load–displacement curves.

**Figure 4:**
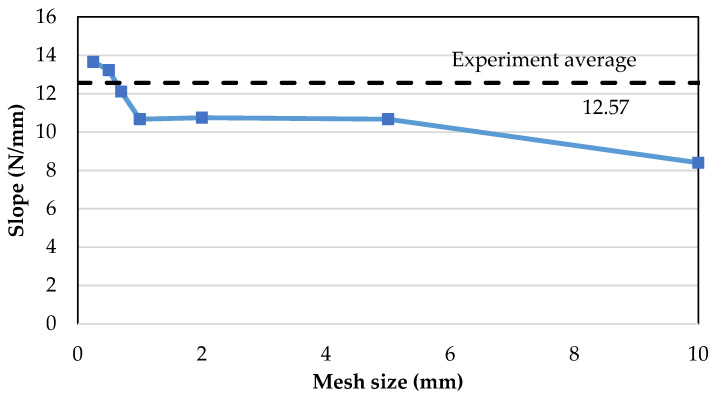
Convergence from coarser to a finer mesh.

Figure 3 shows the load–displacement results of Mode-I models with different mesh sizes ranging from 10 mm to 0.25 mm compared to the experimental results (dotted marker). A noticeable convergence of slope from the coarser size of 10 mm to the finer mesh size of 0.5 mm is visually seen. Mesh refinement from coarse to fine mesh increases the slope to match the experimental gradient and, at the same time, the peak load decreases from exceedingly high towards the average experimental peak load. Such a trend converging towards the experimental curves indicates the feasibility of the current mesh convergence method, by comparing load–displacement results. The curves illustrate that mesh sizes above 2 mm are way off bound in both exceedingly low slope and high peak loads. While mesh sizes of 0.7 and 1 mm have peak loads that are comparable with the experimental data, both have slopes which are still comparably low. At the mesh size of 0.5 mm and smaller, the slope coincides with that of the experiment, where 0.25 mm does not exhibit much improvement on 0.5 mm. The convergence from coarser to a finer mesh by comparing the slope value is distinctly illustrated in Figure 4, where 0.5 mm matches the required experimental slope value while 0.25 mm overestimates it. Therefore, a mesh size of 0.5 mm is chosen for both the Mode-I and Mode-II FE models. The mesh convergence at mesh size less than 0.5 mm agrees with the findings by Turon et al. [7].

### 4.2. Load–Displacement Curves and Model Validation

**Figure 5:**
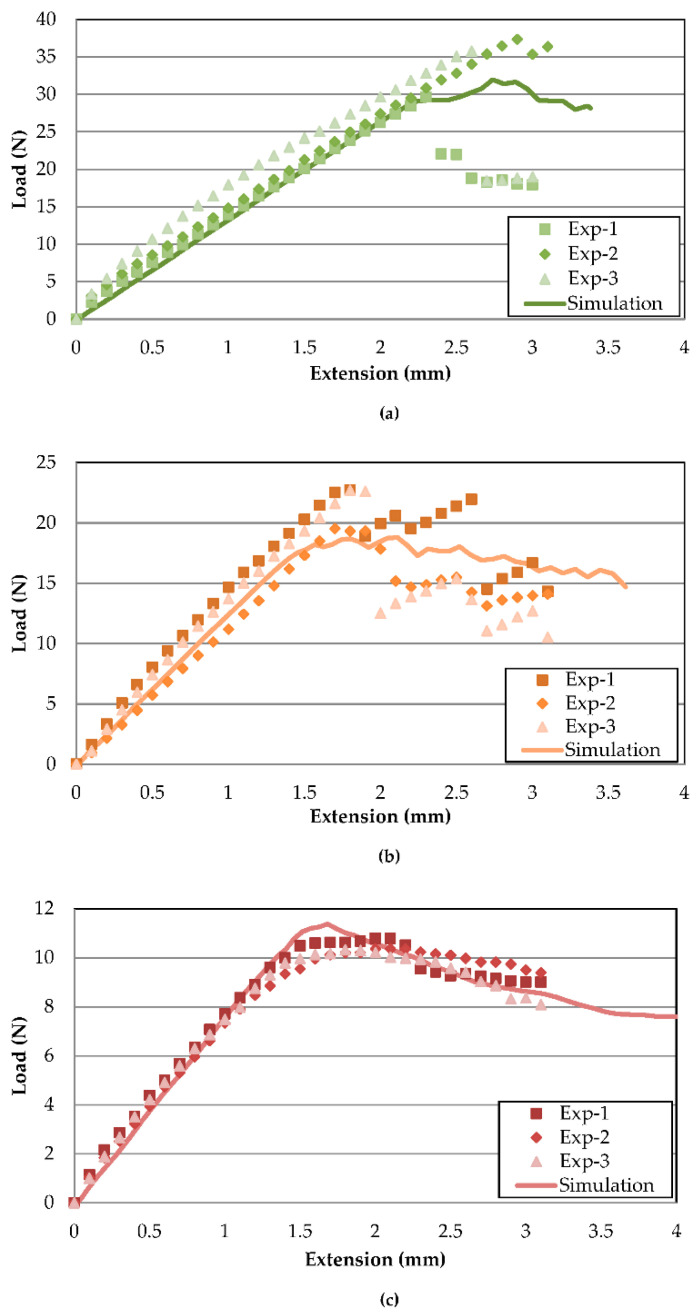
Mode-I load–displacement curve validation between experiment and simulation at (**a**) 30, (**b**) 70 and (**c**) 110 °C.

Figure 5 illustrates the FE model load–displacement validation of Mode-I delamination at temperatures of 30, 70 and 110 °C. The simulation predictions and experimental results coincide with minor errors, showing good agreements between both methods. The stiffness behaviour with relation to temperature is correctly captured from 30 °C to 110 °C. The curve plot can be divided into three main stages. The first stage is the initial increase in load, indicating a build-up of stress across the specimen. As the cohesive element at the crack tip reaches maximum stress, the peak load is reached, where the crack initiates with the first load drop. After an initial load decrease, the occurrence of crack propagation is associated with a general trend of decreasing load. The fluctuation of load after an initial drop is associated with uneven crack propagation, caused by stress concentration build-up and release at the crack tip [33]. Based on experimental results, the increasing temperature has minimal effects on the slope from 30 to 70 °C, similar to findings by Davidson et al. [20]. However, the similarity ends at the highest temperature of 110 °C, with a large drop of the slope in the current study, which is postulated to be caused by different materials. Differences between Mode-I slopes of each temperature are tabulated in Table 5 with a maximum % difference of not more than 6%. 

There is a larger discrepancy between peak loads but, in general, the FE-predicted load curve lies between the fluctuations of the experimental data. For 30 and 70 °C, the experimental results showed sudden large drops of load after the peak, while the drop is more gradual for the finite element models. The abrupt drop during experiments indicates that more energy is accumulated before a sudden release, while, in simulations, the crack grows progressively from element to element. Hence, lower peak loads are predicted for FE. The peak load difference is indicated in Table 5, with 7.41 and 14.76% for 30 and 70 °C, respectively. On the other hand, the 110 °C experimental peak load drops are much smoother compared to the FE counterpart. Therefore, the crack growth region is much better simulated compared to 30 and 70 °C. 

**Figure 6:**
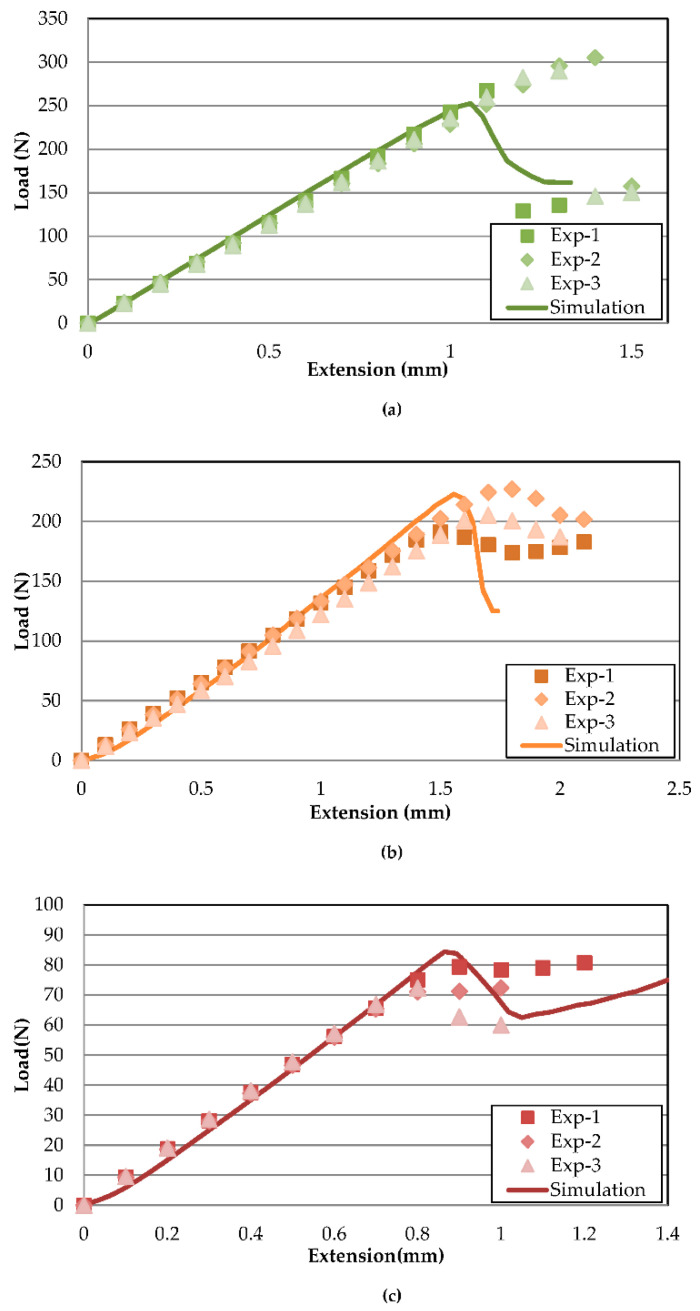
Mode-II load–displacement curve validation between experiment and simulation at (**a**) 30, (**b**) 70 and (**c**) 110 °C.

The load–displacement curves of Mode-II delamination at each temperature are illustrated in Figure 6a–c. Unlike Mode-I, where the load decreases after crack propagation occurs, Mode-II is characterised by load increase after an initial sudden load drop as the crack propagates to the side rollers, preventing any further cracks. Therefore, compared to Mode-I, Mode-II crack initiation does not possess fluctuating loads after a peak load drop. The simulated results appear to agree acceptably with their experimental counterparts, wherein the slope matches with very minor discrepancies. Table 6 shows the corresponding % difference for 30, 70 and 110 °C FE load–displacement curve slopes at 4.12, 7.26 and 3.99%, comparable to the reports by Qin et al. [23] and Gunther et al. [22], despite distinct methods for acquiring penalty stiffness. Thus, this further reaffirms that the penalty stiffness has minor effects on the overall resulting slope validation. For 70 °C, the trend only follows up until the crack initiation, as the load drop is much apparent in the simulation model, whereas the experimental load drop is only up to about 10% before the load increased again.

In similar fashion to Mode-I, the instance of crack initiation is much more difficult to predict, therefore creating a larger divergence between peak loads. The maximum difference in prediction of peak load is for 30 °C, with 15.12%. The larger scatter of peak load is even apparent between the three experimental datasets, only becoming less so at higher temperatures. It should be noted that the FE model slightly overestimates the peak load of 110 °C compared to experimental results for both Mode-I and Mode-II due to a much sharper load drop. The large instantaneous drop of load at lower temperatures could be attributed to brittleness in the composite that leads to a sudden release of the energy during crack propagation. However, for 110 °C, the higher temperatures increased the overall ductility of the adhesive, making crack propagation much smoother [19]. The effects of temperature on the increased ductility and gradual stress release during crack initiation are less well captured in cohesive models. 

### 4.3. Crack Initiation and Stress Distribution

**Figure 7:**
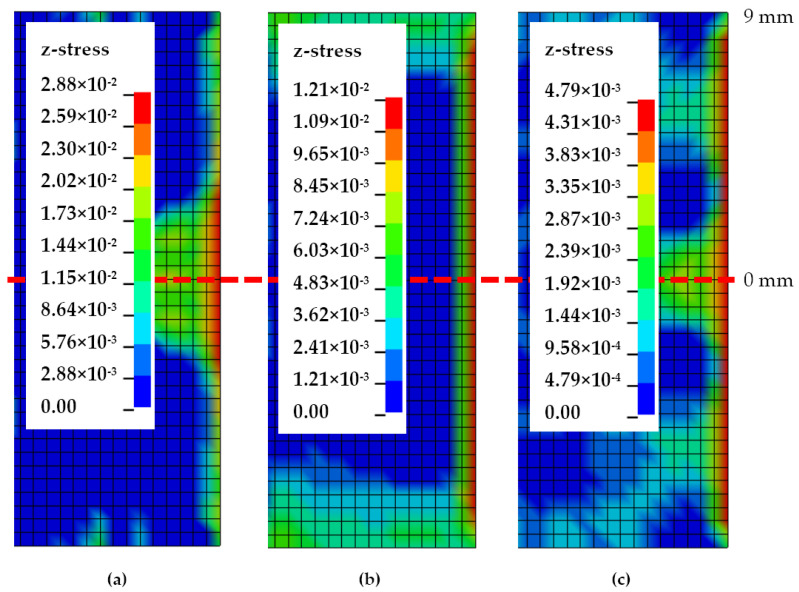
Mode-I crack front stress distribution at (**a**) 30, (**b**) 70 and (**c**) 110 °C.

**Figure 8:**
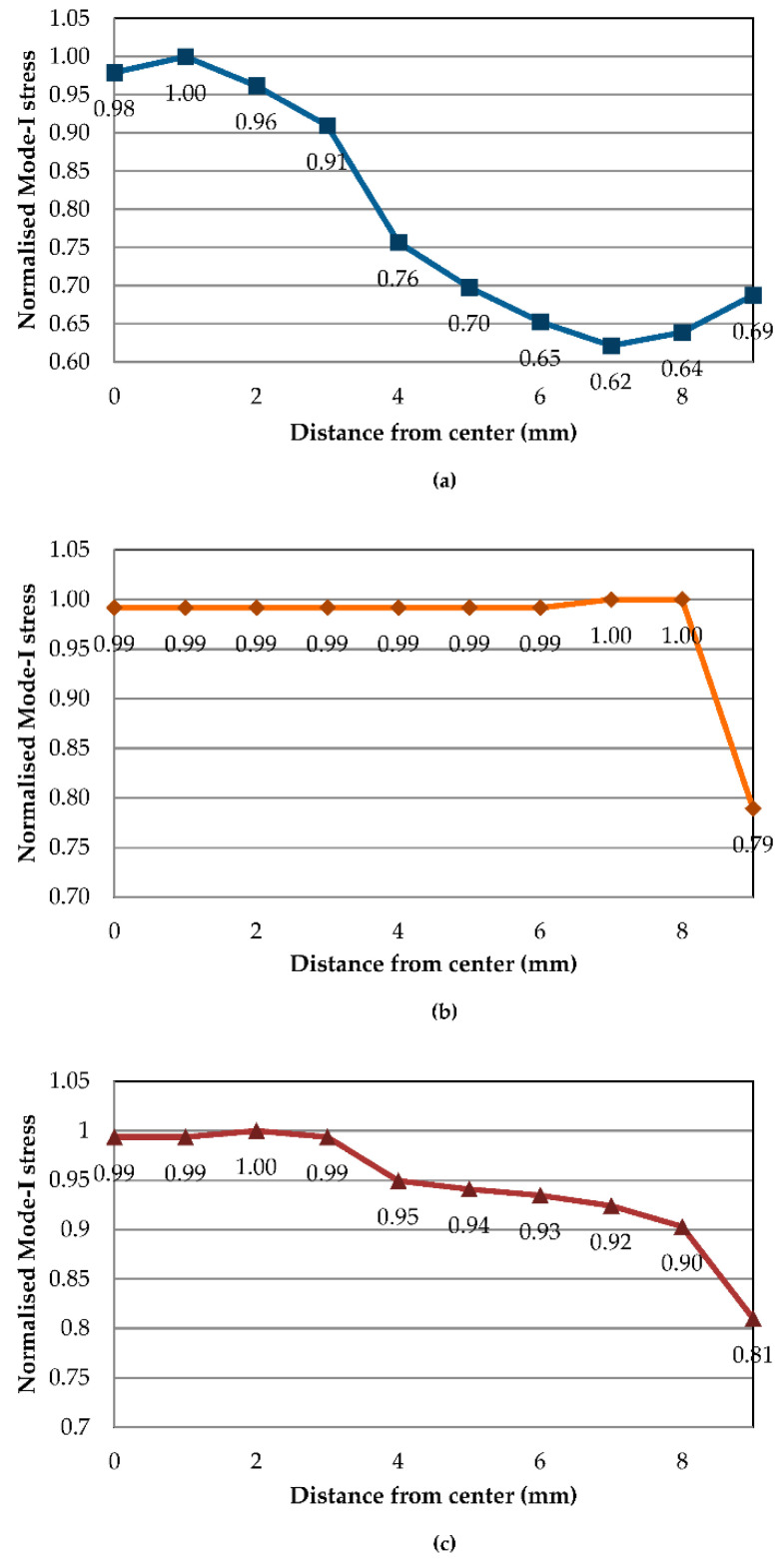
Normalised Mode-I stress distribution along the crack front at (**a**) 30, (**b**) 70 and (**c**) 110 °C.

Figure 7 and Figure 8 demonstrate Mode-I stress distribution across the cohesive delamination width from the DCB FE model, at the maximum stress right before crack initiation. Figure 7 shows the stress contour cut out of cohesive elements near the crack tip, while Figure 8 plots the stress distribution across the crack tip normalised with maximum stress versus the location from the cohesive centre. Due to the FE half model implemented, the stress values are mirrored across the centre. While the stress contours all show the same characteristic of crack initiation from the centre location towards the sides, a substantial change can be noticed by the way stress distribution evolves towards higher temperatures. At 30 °C, elements concentrated at the centre possess much higher stress whilst elements 4 mm and further have much lower stress, about 25% lower than the centre. This stress localisation drastically changes during a temperature of 70 °C where all elements except the outermost (9 mm) from the centre reach maximum Mode-I stress. With the highest temperature of 110 °C, the stress distribution is still akin to that of 70 °C but the stress drops slightly at a distance of 4 to 8 mm (less than 10%). At higher temperatures, stress progression is more rapidly spread from the centre to the outer elements.

**Figure 9:**
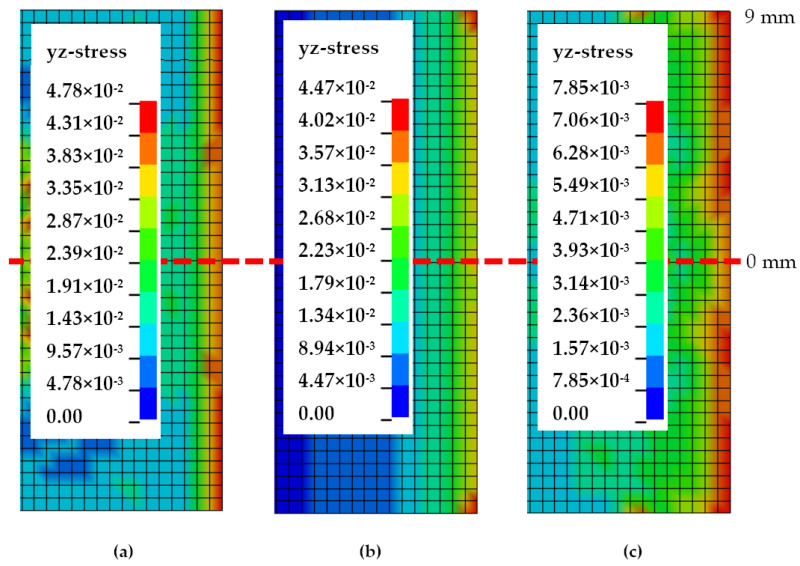
Mode-II crack front stress distribution at (**a**) 30, (**b**) 70 and (**c**) 110 °C.

**Figure 10:**
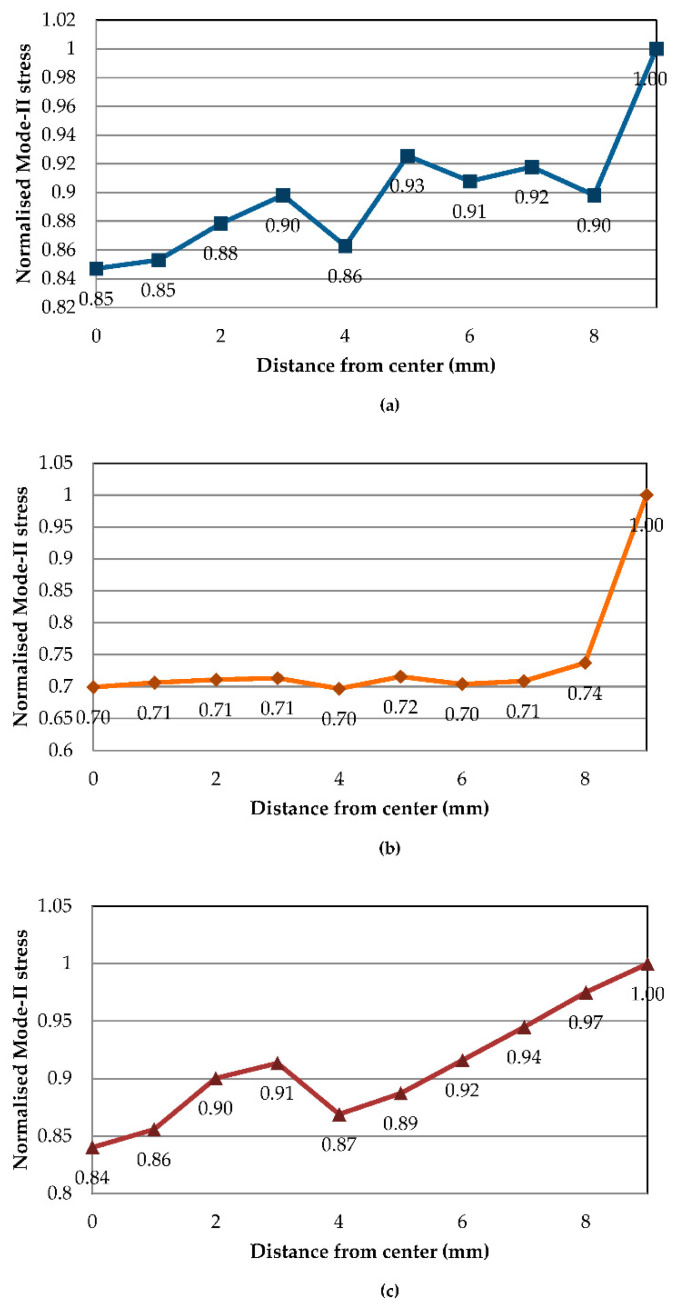
Normalised Mode-II stress distribution along crack tip at (**a**) 30, (**b**) 70 and (**c**) 110 °C.

Crack front stress distributions of Mode-II FE models at temperatures of 30, 70 and 110 °C are exemplified in Figure 9 and Figure 10. Stress contours from Figure 9 show Mode-II typical stress and a crack initiating from the sides of the ENF specimen comparable to analyses by Koloor and Tamin [11]. The corresponding normalised stress values across the crack-initiating front are plotted in Figure 10. At 30 °C, localised normalised stresses are fluctuating more across the crack tip with values of 0.85 to 0.93, while the edge element has significantly higher shear stress. As is with Mode-I, 70 °C Mode-II stress features the trend where all elements from the centremost to an 8 mm distance have the same stress, only contrasted with all shear stresses exhibiting the lowest values, while the elements at 9 mm have substantially higher shear stress by 25%. Such a phenomenon is intriguing as the same behaviour occurs at 70 °C for both modes. On the other hand, Mode-II shear stresses at 110 °C revert to having a much more gradual increase from the centre to the side, with slight fluctuations at 2 and 4 mm. 

## 5. Conclusions

This paper utilises finite element cohesive zone modelling to evaluate temperature effects on Mode-I and Mode-II delamination between aluminium and a GFRP laminated hybrid. Separate temperature-adjusted material models are applied for the aluminium, GFRP and cohesive zone to simulate the effects of temperature to full accuracy. After validation, localised stresses across the crack tips are plotted, where the variation of stress distributions leading to crack initiation are compared between each temperature.

- The validity of the FE model at each temperature is verified with a slope maximum difference within 5.73% for Mode-I at 110 °C and 7.26% for Mode-II at 70 °C.
- Crack front stress is concentrated in the middle for Mode-I, while stress is focused on the sides of Mode-II delamination. Results for 30 °C are characterised by a more fluctuating gradual stress variation for both modes.
- The stress distribution at 70 °C is very polarised, where all elements except the outermost one of Mode-I have practically similar peak stress, while the opposite is observable in Mode-II. A similar trend of more gradual stress variation can be discovered for 110 °C.
- DCB and ENF trends employed from experimental tests successfully obtain temperature-dependent cohesive zone properties.
- A Johnson–Cook material model with temperature dependency and Chang–Chang material model properties at each temperature ensured proper modelling of specimen bending and flexure.
- The validated temperature-dependent cohesive zone model demonstrates the applicability of the current methodology to analyse laminates at high temperatures.

## Figures and Tables

**Table 1:** Material properties of aluminium 2024-T3 for Johnson–Cook material model [29,30,31].

Material Properties	*ρ* (kg/m^3^)	*E* (GPa)	*G* (GPa)	*ν*	
	2869	72.8	27.36	0.33	
Yield surface parameters	*A* (MPa)	*B* (Mpa)	*n*	*C*	*m*
	369	684	0.73	0.0083	1.7
Failure parameters	*d_1_*	*d_2_*	*d_3_*	*d_4_*	*d_5_*
	0.13	0.13	−1.5	0.011	0

**Table 2:** Material properties of glass fibre-reinforced polymer (GFRP) for Chang–Chang failure criterion [28].

	30 °C	70 °C	110 °C
*ν_AB_*	0.321	0.335	0.239
*E_A_* (GPa)	37.12	36.94	30.73
*E_B_* (GPa)	9.75	7.65	2.65
*G_AB_* (GPa)	5.36	3.27	0.13
*X_T_* (MPa)	750.67	719.48	441.77
*Y_T_* (MPa)	58.40	52.88	20.79
*X_C_* (MPa)	816.53	658.42	551.52
*Y_C_* (MPa)	168.51	128.44	94.74
*S_C_* (MPa)	94.93	52.57	6.54
*β*	0.5	0.5	0.5

**Table 3:** Mode-I and Mode-II delamination properties at each temperature [27,28].

	Mode-I			Mode-II		
	30 °C	70 °C	110 °C	30 °C	70 °C	110 °C
*k* (N/mm)	12.57 ± 0.15	12.47 ± 0.46	7.25 ± 0.08	239.97 ± 7.10	134.50 ± 1.91	91.97 ± 2.59
*F_P_* (N)	34.51 ± 2.42	22.04 ± 1.23	10.55 ± 0.16	297.3 ± 4.63	208.32 ± 10.48	74.97 ± 2.34

**Table 4:** Material properties of the cohesive model.

	30 °C	70 °C	110 °C
*E_N_* (GPa)	463.85	460.38	267.52
*E_T_* (GPa)	69.23	38.80	26.53
*G_IC_* (J/m^2^) ^a^	169.50	68.74	27.71
*G_IIC_* (J/m^2^) ^a^	166.70	147.00	28.31
*σ* (MPa)	30.00	12.16	4.90
*τ* (MPa)	45.00	40.09	7.72

^a^ The values are experimentally determined and have been reported in [27,28].

**Table 5:** Mode-I slope and peak load data comparison between experimental and simulation results.

Temperature		30 °C	70 °C	110 °C
*k* (N/mm)	Experiment	12.57	12.47	7.25
	Simulation	13.24	12.46	7.66
Difference (%)		5.38	0.13	5.73
*F_P_* (N)	Experiment	34.51	22.04	10.55
	Simulation	31.95	18.78	11.37
Difference (%)		7.42	14.76	7.83

**Table 6:** Mode-II slope and peak load data comparison between experimental and simulation results.

Temperature		30 °C	70 °C	110 °C
*k* (N/mm)	Experiment	239.97	134.50	91.97
	Simulation	249.85	144.27	95.63
Difference (%)		4.12	7.26	3.99
*F_P_* (N)	Experiment	297.30	208.32	74.97
	Simulation	252.36	222.90	84.36
Difference (%)		15.12	7.00	12.53
